# Supplementary material for: Mutation S115T in IMP-Type Metallo-β-Lactamases Compensates for Decreased Expression Levels Caused by Mutation S119G
Source: Biomolecules. 2019 Nov 11;9(11):724. doi: 10.3390/biom9110724 (PMC6920813; doi:10.3390/biom9110724)
Supplement: Supplementary file 1 [file biomolecules-09-00724-s001.pdf]

biomolecules

Supplementary Materials

Table S1. Agar disc diffusion assay inhibition zone diameters
Means ± standard deviations are given for 4 replicates.

	Zone Diameter (mm)					
β-Lactam	pBCSK(+) *bla*$_{IMP-1}$	pBCSK(+) *bla*$_{IMP-1-S115T}$	pBCSK(+) *bla*$_{IMP-1-S119G}$	pBCSK(+) *bla*$_{IMP-1-S115T-S119G}$	No vector	Empty pBCSK(+)
Ampicillin	0 ± 0	0 ± 0	0 ± 0	0 ± 0	25 ± 1	30 ± 1
Ceftazidime	0 ± 0	0 ± 0	0 ± 0	0 ± 0	38 ± 1	43 ± 3
Imipenem	25 ± 1	25 ± 1	25 ± 1	24 ± 1	33 ± 2	38 ± 2
Meropenem	24 ± 1	25 ± 1	24 ± 1	23 ± 1	39 ± 1	45 ± 3

biomolecules

MDPI

Table S2. Enzyme kinetic constants
Means ± standard deviations of three measurements are reported.

	IMP-1			IMP-1-S115T			IMP-1-S119G			IMP-1-S115T-S119G		
	k_{cat} (s^{-1})	K_m (μM)	k_{cat}/K_m ($\mu M^{-1}s^{-1}$)	k_{cat} (s^{-1})	K_m (μM)	k_{cat}/K_m ($\mu M^{-1}s^{-1}$)	k_{cat} (s^{-1})	K_m (μM)	k_{cat}/K_m ($\mu M^{-1}s^{-1}$)	k_{cat} (s^{-1})	K_m (μM)	k_{cat}/K_m ($\mu M^{-1}s^{-1}$)
**PEN**	2000 ± 100	420 ± 40	4.7 ± 0.1	740 ± 50	600 ± 50	1.2 ± 0.1	900 ± 40	270 ± 20	3.4 ± 0.1	550 ± 10	230 ± 10	2.3 ± 0.1
**AMP**	260 ± 20	340 ± 50	0.77 ± 0.07	140 ± 10	270 ± 30	0.5 ± 0.2	300 ± 40	290 ± 60	1.0 ± 0.1	113 ± 3	190 ± 10	0.59 ± 0.02
**CEF**	122 ± 1	2.1 ± 0.1	57 ± 1	64 ± 1	2.5 ± 0.1	26 ± 1	48 ± 1	1.9 ± 0.1	25 ± 1	22 ± 1	1.7 ± 0.1	13 ± 1
**FOX**	21 ± 1	1.2 ± 0.1	17 ± 1	13 ± 1	1.6 ± 0.1	8.1 ± 0.1	12 ± 1	1.3 ± 0.1	9.6 ± 0.6	10 ± 1	1.1 ± 0.1	9.5 ± 0.2
**CTX**	21 ± 2	1.5 ± 0.1	13 ± 1	19 ± 1	2.6 ± 0.1	7.3 ± 0.3	32 ± 1	4.7 ± 0.3	6.9 ± 0.3	23 ± 1	0.99 ± 0.06	23 ± 1
**CAZ**	18 ± 1	50 ± 6	0.35 ± 0.02	14 ± 1	43 ± 2	0.32 ± 0.01	15.4 ± 0.2	35 ± 1	0.45 ± 0.01	25 ± 2	51 ± 6	0.50 ± 0.03
**IPM**	200 ± 10	33 ± 1	6.1 ± 0.1	153 ± 3	29 ± 1	5.2 ± 0.1	180 ± 3	12 ± 1	15 ± 1	110 ± 13	28 ± 5	4.0 ± 0.3
**MEM**	26 ± 1	7.5 ± 0.6	3.6 ± 0.2	14 ± 1	5.8 ± 0.6	2.3 ± 0.1	54 ± 1	10 ± 1	5.4 ± 0.1	22 ± 1	3.0 ± 0.2	7.5 ± 0.4
**DOR**	180 ± 20	12.8 ± 0.6	14 ± 1	113 ± 7	18 ± 2	6.3 ± 0.3	350 ± 20	29 ± 1	12.1 ± 0.4	250 ± 40	29 ± 8	9 ± 1
**ATM**	ND			ND			ND			ND		
